# Gemella Haemolysans Infective Endocarditis in a Patient With Febrile Neutropenia

**DOI:** 10.7759/cureus.24076

**Published:** 2022-04-12

**Authors:** Logan J Eslinger, Taha Ahmed

**Affiliations:** 1 Neurology, University of Kentucky College of Medicine, Lexington, USA; 2 Internal Medicine, University of Kentucky College of Medicine, Lexington, USA

**Keywords:** gemella haemolysans, transthoracic echocardiogram, acute myeloid leukemia, febrile neutropenia, infective endocarditis

## Abstract

*Gemella* *haemolysans (G. haemolysans)* is a rare cause of native valve infective endocarditis in hospitals and the community. Endocarditis from this species has mostly been reported in patients with congenital or valvular heart disease, recent dental procedures, or underlying gastrointestinal malignancy. We present a case of a 63-year-old male with a history of myelodysplastic syndrome and recent transformation into acute myeloid leukemia with pancytopenia hospitalized to receive induction therapy. While receiving chemotherapy, he developed febrile neutropenia and was found to have bacteremia and evidence of infective endocarditis caused by G.* haemolysans*. We emphasize the importance of suspicion of *G. haemolysans* in immune-compromised patients, as well as outlining the guidelines on appropriate antimicrobial therapy.

## Introduction

*Gemella* is a genus of bacteria most commonly found within the human oropharynx, genitourinary system, and gastrointestinal system [1]. Its incidence in causing bacteremia is increasing due to increased awareness. Very rarely, it can cause infective endocarditis (IE) in hospitals and the community. Specifically, *Gemella morbillorum (G. morbillorum)* is the most reported subtype, with the second being *G. haemolysans *[1]. Other subspecies include *G. bergeriae, G. sanguinis, G. palaticanis*, and *G. cuniculi *[1]. Endocarditis caused by *Gemella* species is usually limited to patients with congenital or valvular heart disease, recent dental procedures, or underlying gastrointestinal malignancy [1-3]. Herein, we report a case of *G. haemolysans* IE in an immune-compromised individual in the setting of neutropenia and active chemotherapy treatment.

## Case presentation

A 63-year-old male with poor dental hygiene presented to the emergency department with a one-month history of worsening fatigue, generalized aches, and malaise, with acute onset dyspnea on exertion when getting out of the car. He had a past medical history of depression and myelodysplastic syndrome (MDS), which recently transformed into acute myeloid leukemia (AML) with MDS-related changes. On presentation, he had stable vital signs and denied fever, chills, nausea, vomiting, diarrhea, constipation, abdominal pain, cough, shortness of breath, chest pain, palpitations, lower extremity edema, oral ulcers, bleeding, or bruising. However, he did endorse pain and aches in his bones.

Due to his history of MDS, he was being evaluated for a stem cell transplant. However, he received an outpatient bone marrow biopsy and repeated complete blood count the day before the presentation, revealing an abnormally elevated blast count at 19%. A colonoscopy one year ago demonstrated diverticulosis but no evidence of any gastrointestinal malignancy. A recent outpatient transthoracic echocardiogram revealed no structural or functional heart abnormalities with a normal left ventricular ejection fraction (LVEF) of > 55%. During hospitalization, he had a peripherally inserted central catheter (PICC) placed for initiation of chemotherapy.

A hematology/oncology consult resulted in him being initiated on cytarabine 100 mg/m^2^ for seven days and daunorubicin 60 mg/m^2^ for three days as induction chemotherapy. He tolerated the first cycle of chemotherapy well, but repeat bone marrow biopsies on days 15 and 22 post-induction showed increasing blast counts of 20% and 31%, respectively, indicating an inadequate response. As part of the trial criteria, he was no longer eligible to continue with the second cycle. He was subsequently placed on re-induction decitabine and venetoclax therapy. 

**Figure 1:**
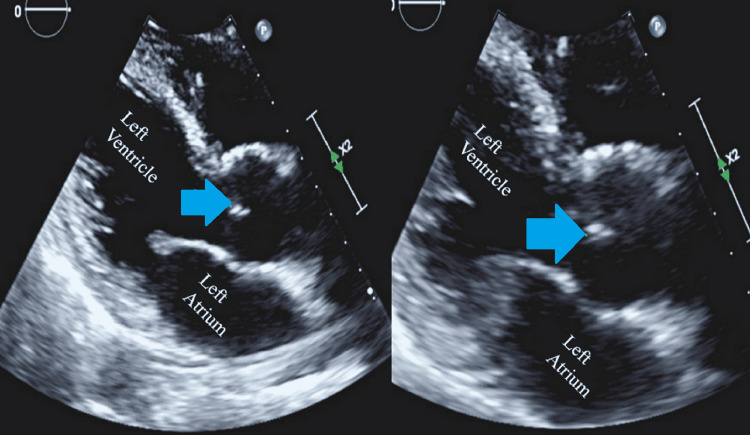
Transthoracic echocardiogram in parasternal long axis view showing vegetation on the right coronary cusp of the aortic valve (blue arrows)

On hospital day 24, the patient developed a persistent fever. Physical examination revealed poor dental hygiene, no rubs/gallops or murmurs on cardiac auscultation, no shortness of breath, no cough, no abdominal pain, and no skin rash. His white blood cell (WBC) count around that time was 0.53 k/uL (normal 3.7 to 10.3 k/uL) with an absolute neutrophil count of 0.02 k/uL (normal 1.6 to 6.1 k/uL). Blood cultures were drawn from the PICC, and a peripheral line grew gram-positive cocci in clusters at 14 hours, which were subsequently positive for *G. haemolysans*. Further results showed susceptibility to vancomycin, ceftriaxone, and penicillin G. Susceptibility to cefepime could not be determined due to insufficient growth. A panorex did not reveal any periapical lucency or abscess. A transthoracic echocardiogram (TTE) was consistent with aortic valve vegetation of 9 mm on the right coronary cusp (Figure 1).

He was initiated on intravenous vancomycin and cefepime, which was later switched to intravenous penicillin G with intravenous gentamycin for synergistic effects. His subsequent blood cultures on hospital day 26 showed no growth, and he completed a total of four weeks of parenteral antibiotics for treatment of *G. haemolysans* infective endocarditis.

The patient was clear of infection from blood cultures before discharge. Intravenous gentamicin therapy was stopped after 15 days due to acute kidney injury. A repeat TTE performed the day before discharge was negative for vegetations. He continued to have pancytopenia due to chemotherapy and required intermittent blood and platelet transfusions. The patient was discharged on oral antimicrobials for neutropenic prophylaxis. On a one-week outpatient follow-up, the patient was doing well and denied any fever.

## Discussion

This report describes a rare occurrence of *G. haemolysans* infective endocarditis (IE) in a patient with febrile neutropenia. When performing a literature review of reported cases of *G. haemolysans* IE, special attention was placed on whether the patients were immune-compromised or suppressed at the time of diagnosis. In addition, the other species of *Gemella *were filtered out of the search to better focus the discussion on the recognition and treatment of *G. haemolysans *specifically. A review of the literature published recently outlined trends in *Gemella *spp IE, showing dental procedures as the most common risk factor (73%), with the mitral valve being the most likely to be involved (39.1%), and *G. morbillorum* being the most common species detected [1]. This article has a broader scope of focus, outlining trends in all known species of *Gemella*. They reported 66 total cases of IE, with only 17 caused by *G. haemolysans* [1]. All the reported cases were found to fit into the scheme of patients predisposed to IE from valvular heart disease, recent dental procedures, or underlying gastrointestinal (GI) malignancy [1]. There is also a case published of a patient with hemochromatosis who developed *G. haemolysans* IE; however, they were also found to have aortic valve insufficiency [4]. 

There is a lack of published evidence of IE caused by *G. haemolysans* in patients that do not possess other common risk factors for infection. This poses a risk of patients receiving late diagnosis and treatment for *G. haemolysans* IE, ultimately increasing mortality rates if surgical intervention is required. Identification of rare species of bacteria on cultures continues to complicate diagnosis in hospitals and clinics without the proper DNA sequencing techniques [2]. It was found that 16s rRNA sequencing is the best method of identifying *Gemella *spp, since, without this, it is often misidentified as *Streptococcus, Leuconostoc, Abiotrophia, Granunicatella,* and *Abiotrophia* species [2]. In our case, the patient had no history of recent dental procedures, with a recent colonoscopy showing no evidence of GI malignancy and a transthoracic echocardiogram (TTE) showing no structural heart abnormalities. There was then little suspicion for endocarditis caused by *Gemella* spp. Advanced laboratory techniques present at the institution allowed for prompt identification of culture and treatment of an infection that otherwise could have progressed to damage of the valvular apparatus, necessitating surgical repair.

Current American Heart Association guidelines outline a preferred treatment regimen for native valve endocarditis infections of streptococcal-like organisms such as *Gemella* spp. It is recommended to start aqueous penicillin G (18-30 million units per 24 hours IV continuously or in six doses over 4-6 weeks) combined with gentamicin (3mg/kg ideal body weight in 2-3 doses over 4-6 weeks) [5]. Our patient received this therapy for four weeks after initial empiric coverage with cefepime and vancomycin. However, our patient did not receive the complete course of intravenous gentamicin due to kidney damage. Careful consideration of the choice of antibiotics is required in patients on active cytotoxic chemotherapy. It is prudent to perform a repeat TTE to ensure the resolution of vegetation with medical therapy.

This case demonstrates the unexpected diagnosis of a rare form of IE in a patient who otherwise does not possess risk factors for *G. haemolysans* bacteremia, highlighting the importance of quick suspicion and treatment when a source or causative organism of bacteremia and IE cannot be identified, especially in hospitals or clinics where advanced DNA sequencing technology is not present.

## Conclusions

*G. haemolysans* IE is most likely to occur in patients with recent dental procedures, structural heart defects, or underlying GI malignancy. In immune-compromised patients who develop IE, *G. haemolysans* should be on the differential diagnosis. Treatment of *G. haemolysans* endocarditis includes a four-week course of aqueous IV penicillin G and gentamicin.
